# Integrative molecular and serum proteomic insights into subclinical *Theileria* infection in captive Fea’s muntjac (*Muntiacus feae*) and red muntjac (*Muntiacus vaginalis*) in Thailand

**DOI:** 10.1016/j.crpvbd.2026.100437

**Published:** 2026-09-16

**Authors:** Pannawat Supapannachart, Sittiruk Roytrakul, Narumon Phaonakrop, Thewarach Vechmanus, Umaporn Maikaew, Pridsana Sreepromma, Karin Hirankailas, Supatsorn Panyalert, Thanapon Chotikaprakal, Sonthaya Tiawsirisup, Kritsada Thongmeesee, Rittidet Kongtawee, Piyanan Taweethavonsawat, Gunnaporn Suriyaphol

**Affiliations:** a Biochemistry Unit, Department of Physiology, Faculty of Veterinary Science, Chulalongkorn University, Bangkok, Thailand; b National Center for Genetic Engineering and Biotechnology (BIOTEC), National Science and Technology Development Agency (NSTDA), Pathum Thani, Thailand; c Chiang Mai Zoo, Zoological Park Organization of Thailand under the Royal Patronage of H.M. the King, Chiang Mai, Thailand; d Khao Kheow Open Zoo, Zoological Park Organization of Thailand under the Royal Patronage of H.M. the King, Chonburi, Thailand; e Department of National Parks, Wildlife and Plant Conservation, Bangkok, Thailand; f Parasitology Unit, Department of Pathology, Faculty of Veterinary Science, Chulalongkorn University, Bangkok, Thailand; g Center of Excellence in Wildlife, Exotic and Aquatic Animal Pathology, Faculty of Veterinary Science, Chulalongkorn University, Pathum Wan, Bangkok, Thailand

**Keywords:** Fea’s muntjac, Northern red muntjac, PCR, Serum proteomics, Subclinical infection, *Theileria*, Wildlife conservation

## Abstract

The Fea’s muntjac (*Muntiacus feae*) and the northern red muntjac (*Muntiacus vaginalis*) are Asian cervids maintained in captive conservation programmes in Thailand, yet information on blood-borne pathogens in these species remains limited. This study aimed to investigate *Theileria* spp. infection in 34 captive muntjacs from four facilities across Thailand using PCR, Sanger sequencing, phylogenetic analysis, and liquid chromatography-tandem mass spectrometry (LC-MS/MS)-based serum proteomics. All animals appeared clinically healthy, and no hemoparasites were observed on blood smears. Nevertheless, *Theileria* spp. DNA was detected by PCR in every individual, indicating subclinical infection. *Babesia* spp. were below the detection limit of the PCR assay, while members of the *Anaplasmataceae* were not detected. Phylogenetic analysis of 15 sequences positioned most isolates among *T. capreoli*-associated (9/15) or other cervid-associated *Theileria* lineages (5/15), while one isolate (1/15) was positioned separately; relationships involving the study isolates were generally weakly supported. Serum proteomics identified 414 *Theileria*-associated proteins consistently detected in both host species. Functional annotation revealed proteins involved in invasion (rhoptry-associated proteins), proteolysis (aspartyl proteases and calpain-like proteins), ubiquitin-mediated regulation, vesicular trafficking, and RNA processing, providing preliminary functional insight into *Theileria*-associated biological processes during subclinical infection. These findings suggest a potential role of captive muntjacs in parasite maintenance where competent vectors are present and demonstrate the value of integrating molecular detection with proteomic profiling to investigate host-parasite interactions in wildlife.

## Highlights

• *Theileria* 18S rRNA was detected by PCR in subclinically infected captive *Muntiacus feae* and *M. vaginalis.*
• Most isolates (9/15) were closely associated with *Theileria capreoli/T.* cf*. capreoli*; others were cervid-associated.
• Serum proteomics identified *Theileria*-associated proteins linked to invasion, proteolysis, and vesicular trafficking.

## Graphical abstract

**Figure:**
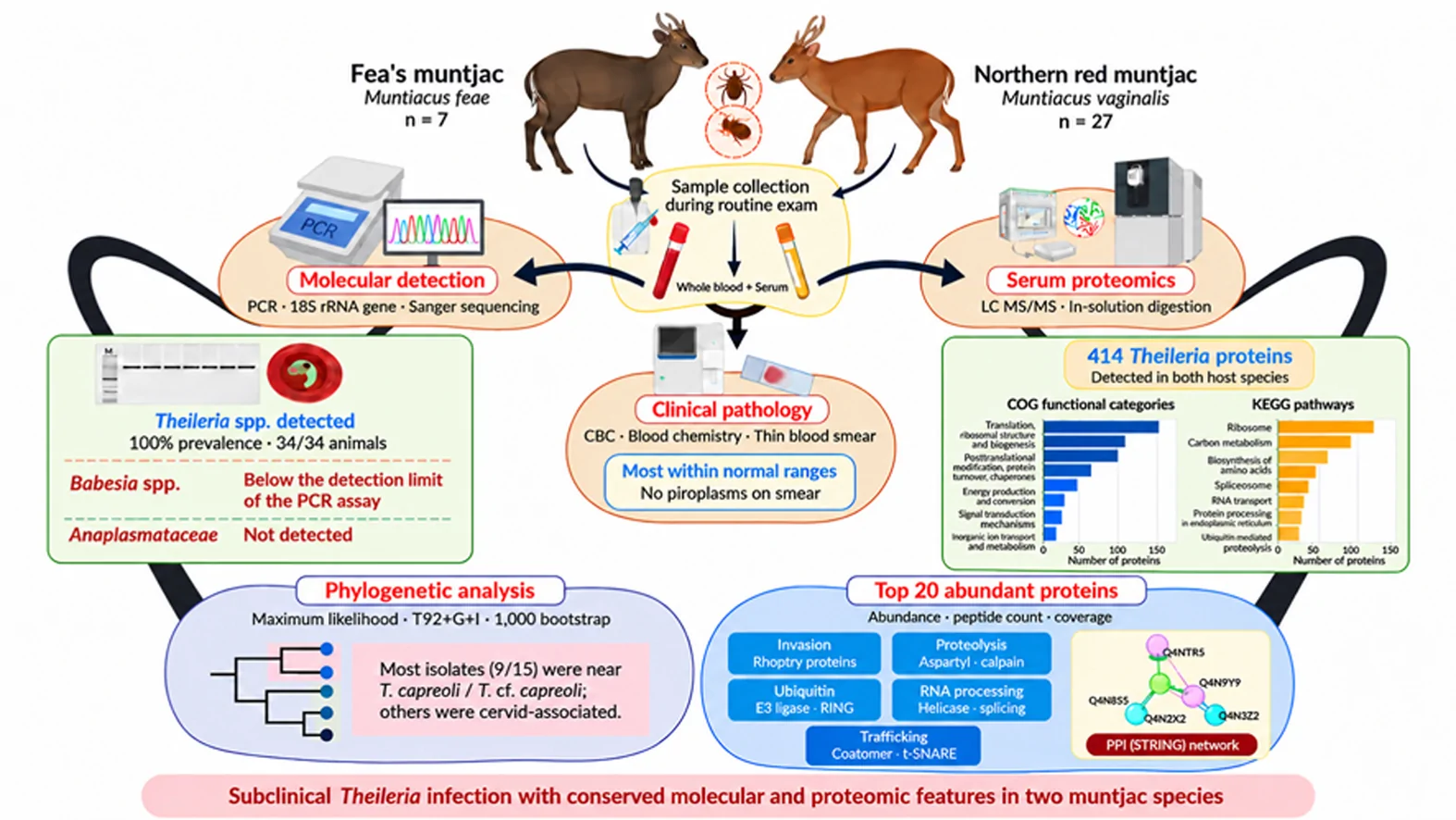

## 1 Introduction

The Fea’s muntjac (*Muntiacus feae*) and the northern red muntjac (*Muntiacus vaginalis*) are Asian cervids inhabiting Southeast Asian forest ecosystems (Francis, 2019). In Thailand, the captive population of *M*. *feae* is critically small (*n* = 7), making the health and long-term management of each individual essential for species conservation. Although *M*. *feae* is currently classified as Data Deficient by the IUCN because of uncertainties regarding its distribution (Timmins et al., 2016a), it is listed as Endangered (EN) in the Thailand National Red List (ONEP, 2020). Recent genomic studies have also improved our understanding of the taxonomy and genetic status of this threatened species, providing an important foundation for future conservation management (Supapannachart et al., 2026a, 2026b). In contrast, *M*. *vaginalis* is more abundant and is classified as Least Concern (LC) by the IUCN despite declining population trends (Timmins et al., 2016b). Recent genomic evidence further demonstrated that all captive red muntjacs examined in Thailand belong to the *M*. *vaginalis* lineage (Supapannachart et al., 2026a).

In captive breeding facilities in Thailand, *M*. *feae* are often maintained in close proximity to *M*. *vaginalis*, creating opportunities for the transmission of blood-borne pathogens between species. Blood-borne pathogens, particularly those of the genera *Theileria*, *Anaplasma*, and *Babesia*, represent important health threats to wild and captive cervids (Dantas-Torres et al., 2012; Koonyosying et al., 2022; Calchi et al., 2025). Among these, *Theileria* spp. are of particular concern due to their widespread occurrence in wild ruminants and their potential for spillover at the wildlife-livestock interface (Morrison et al., 2020; Naveed et al., 2022). *Theileria* spp. infections can cause severe disease in stressed or immunocompromised hosts, particularly in juvenile cervids, with clinical signs including fever, lethargy, and anemia, and may result in high mortality in severe cases (Yabsley et al., 2005; Gieger et al., 2020). However, most *Theileria* infections in cervids across North America, Europe, and East Asia remain subclinical, with infected animals potentially contributing to pathogen maintenance over extended periods (Han et al., 2009; Zanet et al., 2014; Cauvin et al., 2019; Remesar et al., 2019; Gieger et al., 2020). Detection of subclinical *Theileria* spp. infection remains challenging, as conventional blood smear microscopy often lacks the sensitivity required to detect low parasitemia in chronic carriers (Noaman, 2012; Riaz et al., 2023). Consequently, polymerase chain reaction (PCR) targeting the 18S ribosomal RNA gene has become a widely used and highly sensitive method for detecting *Theileria* spp. infections (Altay et al., 2008). PCR-based approaches have identified multiple *Theileria* lineages in cervids across East Asia, including the Reeves’ muntjac (*Muntiacus reevesi*), red deer (*Cervus elaphus*), and sika deer (*Cervus nippon*) in China, as well as Chinese water deer (*Hydropotes inermis argyropus*) in South Korea and sika deer in Japan (Han et al., 2009; Li et al., 2014; Yang et al., 2014; Lee et al., 2019). These findings highlight the widespread distribution of *Theileria* spp. infections in cervids and their implications for wildlife health and conservation. At Khao Kheow Open Zoo, Thailand, *Theileria* DNA has been detected in Eld’s deer (*Rucervus eldii*) and Indochinese serows (*Capricornis sumatraensis*) (Sanyathitiseree et al., 2008). Subsequent surveillance identified *Theileria* spp. in multiple ectoparasites, including the ticks *Rhipicephalus microplus* and *Dermacentor auratus*, and deer keds (*Lipoptena* spp.), together with species of *Anaplasma* and *Babesia* (Tiawsirisup et al., 2023; Sri-in et al., 2024). Despite these findings, *M*. *feae* and *M*. *vaginalis* have not been surveyed for *Theileria* spp. infections in Thailand, representing an important knowledge gap in regional epidemiology and conservation management.

While PCR provides direct molecular evidence of pathogen DNA, it provides limited information on parasite-associated biological processes and host-pathogen interactions (Beltran et al., 2017). In this context, proteomic approaches, particularly liquid chromatography-tandem mass spectrometry (LC-MS/MS), provide a powerful complementary strategy for identifying pathogen-associated proteins expressed during infection and characterizing their biological functions (Lee and Kim, 2022). Additionally, proteomic methodologies have been successfully applied to the investigation of parasite biology, host responses, and biomarker discovery in blood-borne pathogen infections, underscoring the value of serum proteomics for investigating complex host-parasite interactions (Lin et al., 2011; Pumpitakkul et al., 2024). Accordingly, this study aimed to investigate *Theileria* infection in *M*. *feae* and *M*. *vaginalis* in Thailand, examine the phylogenetic relationships of the detected *Theileria* spp. sequences, and characterize parasite-associated proteins using an integrated approach comprising PCR, DNA sequencing, and in-solution serum LC-MS/MS proteomics.

## 2 Materials and methods

### 2.1 Animals

A total of 34 adult animals, comprising 7 *M*. *feae* and 27 *M*. *vaginalis*, were included in this study. The ages of *M*. *feae* ranged from 3 years to 9 months to 16 years and 3 months. Although the exact ages of *M*. *vaginalis* were unavailable, all were confirmed to be adults based on zoo and wildlife breeding center records. Animals were sampled from four facilities across Thailand: Chiang Mai Zoo (Chiang Mai) and Khao Kheow Open Zoo (Chonburi), both under the Zoological Park Organization of Thailand (ZPOT), as well as Ton Nga Chang Wildlife Breeding Center (Songkhla) and Om Koi Wildlife Breeding Center (Chiang Mai), managed by the Department of National Parks, Wildlife and Plant Conservation (DNP). Detailed information on the individual animals is provided in Supplementary Table S1. All animals were born at their respective facility, except one *M*. *feae* (FM4F), which was acquired from an unrecorded outside source prior to the establishment of the current husbandry records. Across all participating facilities, animals received in-feed ivermectin approximately every four months, and long grass and vegetation were cleared weekly to reduce tick and other vector habitats. Sample collection was performed during routine veterinary examinations. None of the animals exhibited clinical signs commonly associated with blood-borne pathogen infections at the time of sampling, including anemia, jaundice, lethargy, or weight loss, which have been reported in clinically affected cervids and other ruminants (Yabsley et al., 2005; Stuen, 2020).

### 2.2 Sample collection and preparation

Blood samples were collected from jugular or cephalic veins during routine veterinary procedures using EDTA and serum clot activator vacutainer tubes for hematological/molecular analyses and serum preparation, respectively. Serum was obtained by allowing whole blood to clot at room temperature for 30–45 min, followed by centrifugation at 3000× *g* for 15 min at 4 °C. Complete blood count (CBC) and serum biochemistry were performed at the Vet Central Lab (Nonthaburi, Thailand). Thin blood smears were prepared and microscopically examined for hemopathogens following established protocols (Koonyosying et al., 2022). Reference intervals for hematological and blood chemistry parameters were based on available data for *M. vaginalis* in the Zoological Information Management System, due to the absence of species-specific reference values for *M. feae* (ZIMS, 2025).

For molecular analysis, genomic DNA was extracted from 600 μl of whole blood using the Quick-DNA Miniprep Plus Kit (Zymo Research, Irvine, CA, USA) according to the manufacturer’s instructions. DNA concentration and purity were assessed using a Qubit Fluorometer and a NanoDrop One spectrophotometer (Thermo Fisher Scientific, Waltham, MA, USA). Extracted DNA was stored at −20 °C until PCR amplification. For proteomic analysis, serum was supplemented with Halt Protease Inhibitor Cocktail (Thermo Fisher Scientific) at a 1:50 (v/v) ratio and stored at −20 °C until further analysis.

### 2.3 Molecular detection of blood-borne pathogens

Molecular detection of blood-borne pathogens was performed in all sampled animals using conventional PCR assays targeting the 18S rRNA gene of *Theileria* spp. and *Babesia* spp., and the 23S rRNA gene of members of the family *Anaplasmataceae*. Primer sequences, target genes, and expected amplicon sizes are summarized in Table 1. PCR assays for *Theileria* spp. and *Babesia* spp. were performed following the protocol of Sri-in et al. (2024), whereas detection of *Anaplasmataceae* followed Alhassan et al. (2021). PCR amplification was carried out using G-Storm (Gene Technologies, Essex, UK) and BIOER (BIOER Technology, Hangzhou, China) thermal cyclers. Previously confirmed pathogen-positive DNA samples were included as positive controls, while nuclease-free water was used as the no-template negative control in each PCR run. PCR products were separated by 1% (w/v) agarose gel electrophoresis in 1× TAE buffer, stained with Prime Juice DNA staining dye (Bio-Helix, New Taipei City, Taiwan), and visualized under ultraviolet illumination using a ChemiDoc imaging system (Bio-Rad Laboratories, Hercules, CA, USA).

**Table 1:** PCR primers and target genes used for detection of blood-borne pathogens.

Target taxon	Gene	Primer	Primer sequence (5′–3′)	Amplicon size (bp)	Reference
*Theileria* spp.	18S rRNA	989-F	AGTTTCTGACCTATCAG	1098	Allsopp et al. (1993)
		990-R	TTGCCTTAAACTTCCTTG		
*Babesia* spp.	18S rRNA	Bab-F	GTTTCTGMCCCCATCAGCTTGAC	420–440	Hilpertshauser et al. (2006)
		Bab-R	CAAGACAAAAAGCTGCTTGAAAC		
*Anaplasmataceae*	23S rRNA	A-23sF2	TCGATGGGAATCAGGTTAATATTCCTG	830	Alhassan et al. (2021)
		A-23sR2	TTGTACTATAAAAGCTGATTTCCG		

### 2.4 Sequencing and phylogenetic analysis

PCR products showing clear bands of the expected size were excised from the gel and purified using the QIAquick Gel Extraction Kit (Qiagen, Hilden, Germany) according to the manufacturer’s instructions. Purified PCR products were subjected to Sanger sequencing (ATCG, Pathum Thani, Thailand). Consensus nucleotide sequences were generated from forward and reverse Sanger sequencing reads using CodonCode Aligner version 13 (CodonCode Corporation, Centerville, MA, USA). Sequence orientation was verified by BLASTn comparison with homologous sequences in GenBank, and reverse-complement sequences were generated when necessary to ensure that all sequences were oriented in the 5′–3′ direction (Altschul et al., 1990). Reference sequences of *Theileria* spp. were retrieved from GenBank, and *Babesia capreoli* (GenBank: FJ944828.1) was included as the outgroup. The study and reference sequences were aligned using the ClustalW algorithm implemented in MEGA 12 (Kumar et al., 2024). The alignment was visually inspected, and poorly aligned and incompletely covered regions at the 5′- and 3′-ends were manually trimmed using CodonCode Aligner, resulting in a final alignment of 492 bp. The best-fit nucleotide substitution model was determined using the maximum-likelihood model selection procedure in MEGA 12. Phylogenetic relationships were reconstructed using the maximum-likelihood method under the Tamura 3-parameter model with gamma-distributed rate variation and a proportion of invariant sites (T92+G+I). The robustness of the tree topology was assessed using 1000 bootstrap replicates. The phylogenetic tree was rooted with *B. capreoli* as the outgroup.

### 2.5 In-solution serum protein digestion and LC-MS/MS analysis

Protein samples (5 μg per sample) were reduced with dithiothreitol (DTT), alkylated with iodoacetamide (IAA), and digested with sequencing-grade modified trypsin (Promega, Madison, WI, USA) in 10 mM ammonium bicarbonate using a standard in-solution digestion protocol. The resulting peptides were acidified with 0.1% formic acid and stored at −20 °C until LC-MS/MS analysis. Peptides (1 μl) were analyzed using an Ultimate 3000 Nano/Capillary LC system (Thermo Fisher Scientific) coupled to a ZenoTOF 7600 mass spectrometer (SCIEX, Marlborough, MA, USA). Peptides were separated on a C18 reverse-phase analytical column using a 5–55% acetonitrile gradient over 30 min at a flow rate of 0.30 μl/min. Mass spectrometry was performed in positive ion mode using data-dependent acquisition (Top 50). Each sample was analyzed in technical triplicate.

### 2.6 Data processing, statistical analysis, and bioinformatics

Raw LC-MS/MS data were processed using MaxQuant with the integrated Andromeda search engine (Tyanova et al., 2016). MS/MS spectra were searched against the UniProt *Theileria* database (taxon ID: 5874) with trypsin specified as the proteolytic enzyme. A maximum of two missed cleavages was allowed, and the precursor mass tolerance was set to 20 ppm. Carbamidomethylation of cysteine residues was defined as a fixed modification, whereas methionine oxidation and protein N-terminal acetylation were specified as variable modifications. Matching between runs was enabled to improve peptide and protein identification across samples. Protein identification was filtered at a false discovery rate (FDR) of 1% using a reverse-sequence decoy strategy, with a maximum of five modifications permitted per peptide. All other parameters were maintained at their default settings.

Label-free protein abundance was assessed using log_2_-transformed peptide ion intensities. Proteins lacking functional annotation in the reference database were excluded from further analysis. To ensure reliable protein detection, proteins were retained only if they were identified in at least 50% of individuals within each host group (i.e. ≥ 4/7 for *M*. *feae* and ≥ 15/27 for *M*. *vaginalis*), and only proteins detected in both host species were retained for subsequent analyses (Lazar et al., 2016; Tyanova et al., 2016). Missing values were treated as zero values during ranking analyses. To identify highly abundant proteins, the mean log_2_ intensity for each protein was calculated across all samples from both *M*. *feae* and *M*. *vaginalis*. Proteins were then ranked according to mean log_2_ intensity, number of supporting peptides, and sequence coverage. The top 20 proteins were selected for further characterization. Data processing and ranking were performed using custom scripts in R (v.4.3.0). Functional annotation was conducted using the eggNOG database to assign proteins to Clusters of Orthologous Groups (COG) functional categories and Kyoto Encyclopedia of Genes and Genomes (KEGG) pathways (Cantalapiedra et al., 2021). Gene Ontology (GO) annotations retrieved from UniProtKB/Swiss-Prot were used to further characterize the biological functions of the identified proteins. Functional and pathway distributions were visualized using bar plots generated with the *ggplot2* package in R (v.4.3.0).

To explore functional relationships among the identified *Theileria* spp. proteins, protein-protein interaction (PPI) network analysis was performed on the top 20 abundant proteins. Because the identified proteins originated from multiple *Theileria* species, each protein was mapped to its closest ortholog in *Theileria parva* for STRING-based PPI analysis using the UniProt similarity search function. Proteins without identifiable orthologs were excluded from downstream analyses. The resulting *T. parva* ortholog identifiers were analyzed using the STRING database (v.12.0; taxonomy ID: 5875) using a minimum interaction confidence score of 0.400 and all interaction sources enabled (Szklarczyk et al., 2023).

## 3 Results

### 3.1 Hematological profiles

The hematological and blood chemistry profiles of all 34 muntjacs were determined (Supplementary Table S2). Most hematological and blood chemistry values were within the reference intervals for *M. vaginalis*, and no hemopathogens were detected by thin blood smear examination.

### 3.2 Molecular detection of blood-borne pathogens

PCR targeting the 18S rRNA gene detected *Theileria* spp. DNA in all examined animals (34/34), whereas *Babesia* spp. were below the detection limit of the PCR assay, and *Anaplasmataceae* were not detected in any of the examined animals. Representative PCR results are shown in Supplementary Fig. S1.

### 3.3 Sequencing and phylogenetic analysis

Of the *Theileria*-positive samples, 19 with PCR products of sufficient quality and quantity were subjected to Sanger sequencing, of which 15 yielded reliable sequences and were therefore included in the phylogenetic analysis. MJ19M was excluded because conflicting forward/reverse sequencing signals were consistent with a suspected mixed infection, whereas FM4F, MJ15F, and MJ32F were excluded because insufficient sequence coverage or forward/reverse read overlap prevented the generation of reliable consensus sequences. A total of 15 *Theileria* sequences, including one isolate from *M. feae* and 14 isolates from *M. vaginalis*, were submitted to the GenBank database under the accession numbers PZ295588-PZ295602. Additionally, the positive control amplicons from the *Babesia* spp. and *Anaplasmataceae* PCR assays were sequenced and showed the highest similarity to *Babesia vogeli* (99.28%) and *Anaplasma platys* (100%), respectively, supporting the specificity and performance of the corresponding assays.

Maximum-likelihood phylogenetic analysis of the partial 18S rRNA sequences revealed genetic heterogeneity among the *Theileria* spp. sequences obtained in this study (Fig. 1). Nine isolates (MJ23M, MJ29F, MJ27F, MJ37F, FM6M, MJ21M, MJ31F, MJ38F, and MJ22M) were positioned among *Theileria* cf*. capreoli* and *T. capreoli* reference sequences. Five isolates (MJ18F, MJ28M, MJ33F, MJ16F, and MJ24F) were positioned among other cervid-associated *Theileria* lineages. MJ17M was positioned separately from the other study isolates, with no bootstrap support for its placement. Overall, relationships involving the study isolates were weakly supported, whereas several reference lineages showed moderate to strong bootstrap support.

**Fig. 1:**
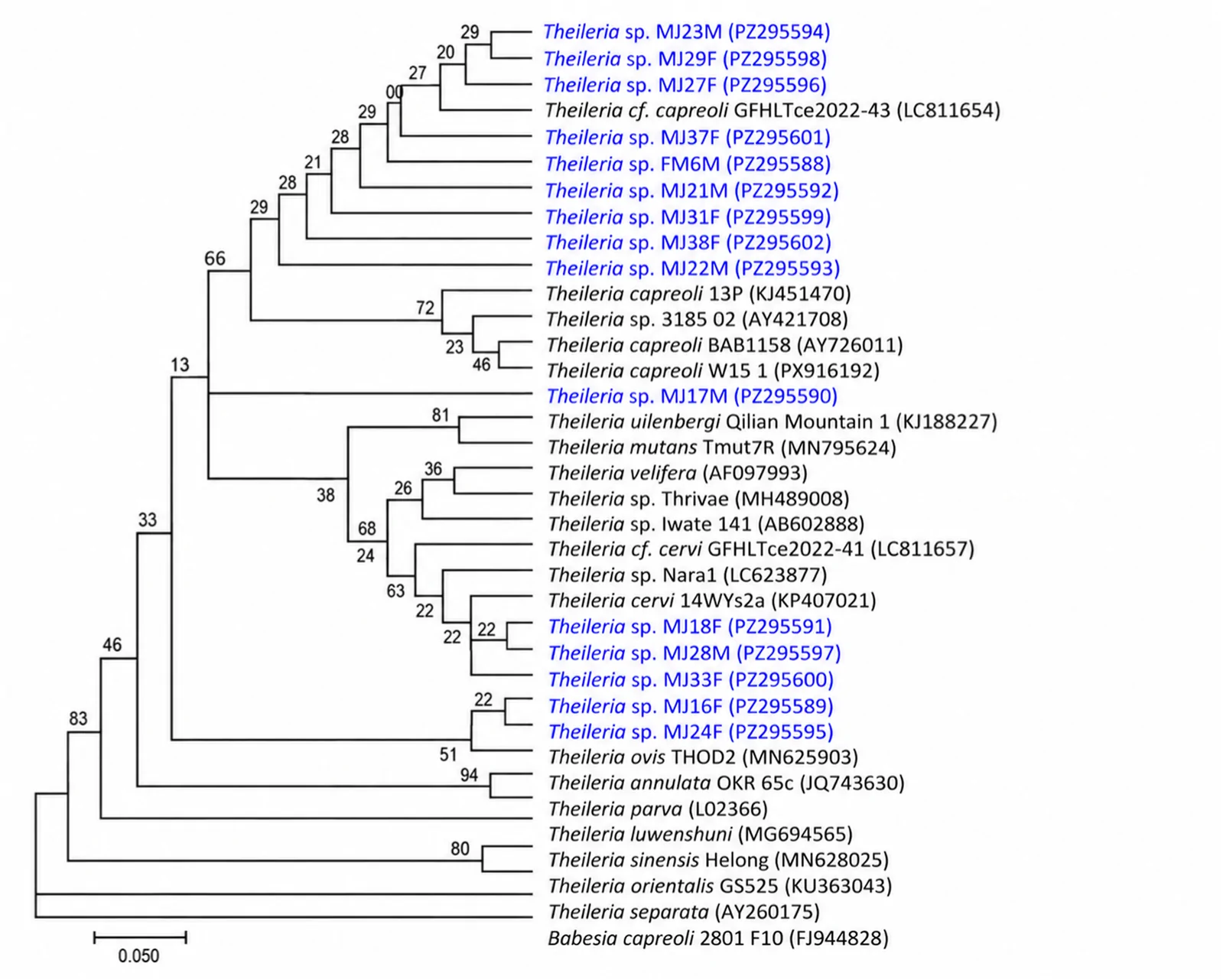
Maximum-likelihood phylogenetic tree based on partial 18S rRNA gene sequences of *Theileria* spp. detected in the Fea’s muntjac (*Muntiacus feae*) and the northern red muntjac (*Muntiacus vaginalis*). Sequences obtained in this study are highlighted in blue, whereas reference sequences retrieved from the GenBank database are shown with their accession numbers and isolate codes. The tree was constructed using the maximum likelihood method under the T92+G+I model with 1000 bootstrap replicates. *Babesia capreoli* was included as the outgroup. The scale-bar represents the number of nucleotide substitutions per site.

Pairwise nucleotide sequence identity analysis revealed 99.79–100% identity among the nine isolates positioned among *T. capreoli/T.* cf*. capreoli* reference sequences and 99.39–99.79% identity between these isolates and their closest *T. capreoli*/*T.* cf*. capreoli* references. The five isolates positioned among other cervid *Theileria* lineages shared 99.38–100% identity with one another and 98.35–98.76% identity with their closest GenBank references. MJ17M showed 98.76% identity to its closest reference, *T.* cf*. capreoli* (GenBank: LC811654). Sequence identity between the two sets of isolates ranged from 97.53% to 97.94% (Supplementary Table S3).

### 3.4 Identification of *Theileria* spp. proteins in *M*. *feae* and *M*. *vaginalis* serum

Serum proteomic analysis identified 6886 *Theileria*-associated proteins in *M*. *feae* and 12,237 in *M*. *vaginalis*, with 6086 detected in both host species. After excluding proteins lacking functional annotation, 4399 proteins remained in *M*. *feae* and 7802 in *M*. *vaginalis*, including 3877 shared proteins. Application of the detection frequency criteria identified 414 proteins consistently detected in both host species, which were retained for downstream functional analyses. COG functional classification revealed that these proteins were distributed across multiple functional categories. The largest proportion of proteins was assigned to the “function unknown” category. Among the annotated categories, proteins involved in post-translational modification, protein turnover, and chaperones were the most abundant. Other highly represented categories included translation, RNA processing and modification, replication and repair, signal transduction mechanisms, and transcription (Fig. 2A). KEGG pathway analysis identified proteins associated with several cellular pathways. Metabolic pathways represented the largest KEGG category, followed by spliceosome, ribosome biogenesis in eukaryotes, purine metabolism, and pyrimidine metabolism (Fig. 2B).

**Fig. 2:**
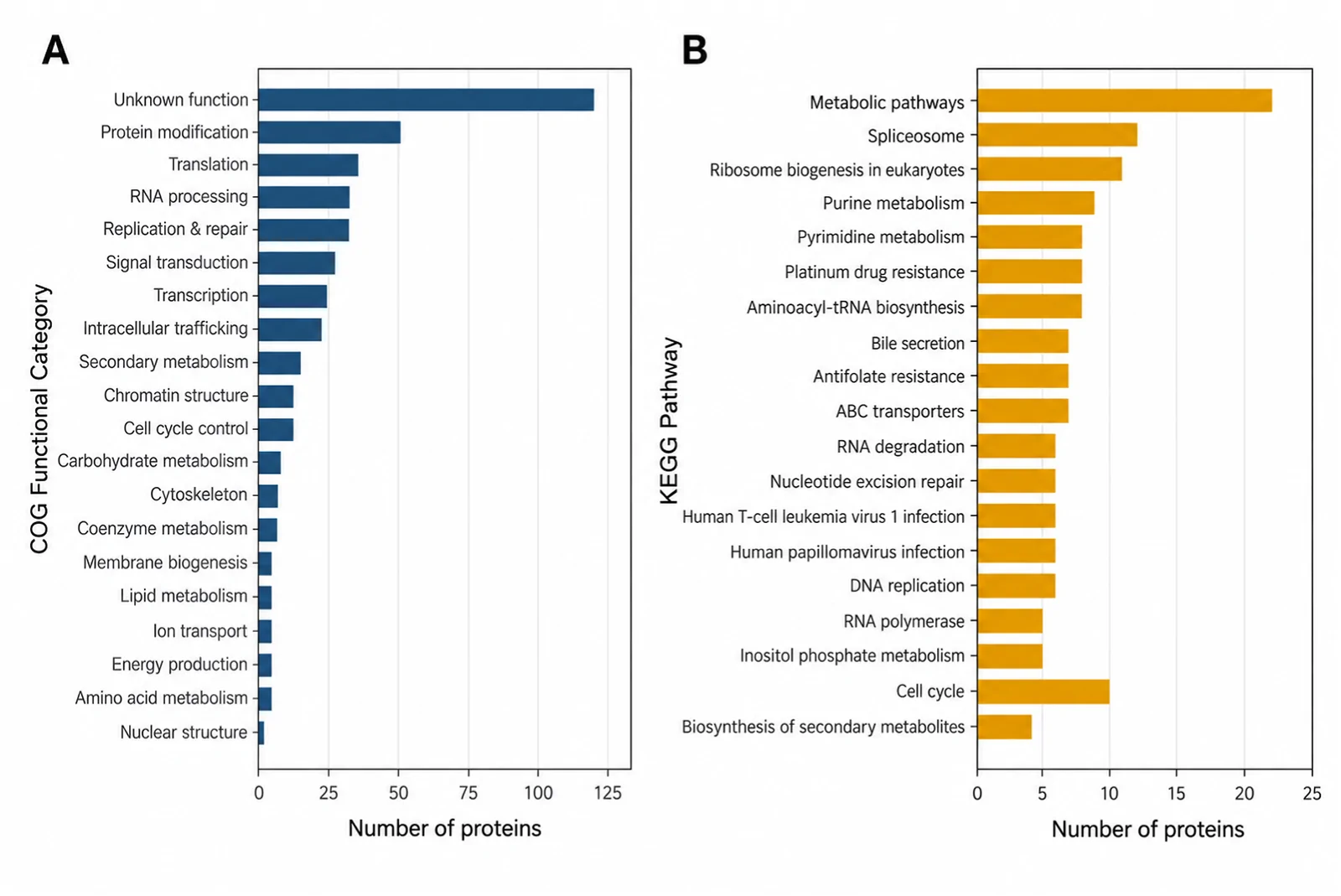
Functional annotation of the 414 shared *Theileria*-associated proteins identified in serum samples from the Fea’s muntjac (*Muntiacus feae*) and the northern red muntjac (*Muntiacus vaginalis*) based on eggNOG analysis. **A** Clusters of Orthologous Groups (COG) functional classification. **B** Kyoto Encyclopedia of Genes and Genomes (KEGG) pathway annotation.

The 20 most abundant shared *Theileria*-associated proteins are summarized in Table 2, whereas all filtered shared proteins are provided in Supplementary Table S4. The top-ranked proteins represented diverse functional categories, including proteolysis, ubiquitin-mediated regulation, gene expression and RNA processing, intracellular transport, signal transduction, and lipid metabolism. Representative proteins included aspartyl protease and calpain catalytic domain-containing proteins, both associated with proteolysis (GO:0006508), E3 ubiquitin-protein ligases and RING-type domain-containing proteins, associated with protein ubiquitination (GO:0016567), lysine-tRNA ligase, pre-mRNA splicing factor 18, and ATP-dependent RNA helicase, with functions related to translation and RNA processing; as well as coatomer subunit gamma and t-SNARE coiled-coil domain-containing proteins, associated with intracellular transport. PNPLA domain-containing proteins, *Sfi*I-subtelomeric related proteins, rhoptry-associated proteins, and several hypothetical membrane proteins were also identified among the top-ranked proteins.

**Table 2:** Top 20 abundant *Theileria*-associated proteins identified in serum samples from *Muntiacus feae* and *Muntiacus vaginalis*.

Rank	UniProt ID	Protein name	Peptide count	Coverage (%)	Log_2_ intensity (mean ± SD)	Biological process (GO)	Molecular function (GO)
1	Q4UD05	Aspartyl protease, putative (EC 3.4.23.-)	6	14.4	15.35 ± 1.51	Protein processing [GO:0016485]	Aspartic-type endopeptidase activity [GO:0004190]
2	A0A976M5G6	Palmitoyltransferase (EC 2.3.1.225)	7	23.1	15.10 ± 1.26	Protein targeting to membrane [GO:0006612]	Protein-cysteine S-palmitoyltransferase activity [GO:0019706]
3	J4C9B2	Coatomer subunit gamma	9	11.5	14.86 ± 0.93	Endoplasmic reticulum to Golgi vesicle-mediated transport [GO:0006888]; intra-Golgi vesicle-mediated transport [GO:0006891]; intracellular protein transport [GO:0006886]; protein secretion [GO:0009306]	Structural molecule activity [GO:0005198]
4	A0A3B0MTQ6	*Sfi*I-subtelomeric related protein family member, putative	49	22.4	14.51 ± 1.63	Not assigned	Not assigned
5	Q4MZX2	Lysine-tRNA ligase (EC 6.1.1.6) (Lysyl-tRNA synthetase)	9	23.0	14.32 ± 1.52	Lysyl-tRNA aminoacylation [GO:0006430]	ATP binding [GO:0005524]; lysine-tRNA ligase activity [GO:0004824]; tRNA binding [GO:0000049]
6	Q4U9F3	E3 ubiquitin-protein ligase (EC 2.3.2.27)	28	15.5	14.27 ± 1.57	Protein ubiquitination [GO:0016567]; ubiquitin-dependent protein catabolic process *via* the N-end rule pathway [GO:0071596]	Ligase activity [GO:0016874]; ubiquitin protein ligase activity [GO:0061630]; zinc ion binding [GO:0008270]
7	Q4N7I3	RING-type domain-containing protein	10	11.5	14.27 ± 1.58	Protein ubiquitination [GO:0016567]; rescue of stalled ribosome [GO:0072344]	Ribosome binding [GO:0043022]; ubiquitin protein ligase activity [GO:0061630]
8	J4DNM4	t-SNARE coiled-coil homology domain-containing protein	5	10.6	14.25 ± 1.58	Vesicle-mediated transport [GO:0016192]	Not assigned
9	L0B3Q4	PNPLA domain-containing protein	10	16.0	13.88 ± 2.92	Lipid homeostasis [GO:0055088]; triglyceride catabolic process [GO:0019433]	Triacylglycerol lipase activity [GO:0004806]
10	A0A976QWJ0	Zinc finger PHD-type domain-containing protein	43	15.0	13.80 ± 2.46	Not assigned	Zinc ion binding [GO:0008270]
11	J4C2X2	Non-specific serine/threonine protein kinase (EC 2.7.11.1)	3	12.8	13.75 ± 1.32	Not assigned	ATP binding [GO:0005524]; protein serine/threonine kinase activity [GO:0004674]
12	L0B128	Calpain catalytic domain-containing protein	5	7.1	13.63 ± 1.44	Proteolysis [GO:0006508]	Calcium-dependent cysteine-type endopeptidase activity [GO:0004198]
13	Q4UI23	RING-type domain-containing protein	9	9.2	13.44 ± 2.53	Protein ubiquitination [GO:0016567]; rescue of stalled ribosome [GO:0072344]	Ribosome binding [GO:0043022]; ubiquitin protein ligase activity [GO:0061630]
14	Q4UAX6	*Theileria*-specific hypothetical membrane protein, putative	24	15.5	13.22 ± 1.43	Not assigned	Not assigned
15	J4CCM7	Pre-mRNA-splicing factor 18	33	24.2	12.99 ± 1.62	Generation of catalytic spliceosome for second transesterification step [GO:0000350]	Not assigned
16	A0A976SKR9	Peroxisome-assembly ATPase (EC 3.6.4.7)	7	23.5	12.97 ± 0.81	Not assigned	ATP binding [GO:0005524]; ATP hydrolysis activity [GO:0016887]
17	Q4UHI3	Tpr-related protein family member, putative	24	18.7	12.91 ± 2.63	Not assigned	Not assigned
18	Q4MYW3	W2 domain-containing protein	18	31.8	12.78 ± 2.01	Not assigned	Not assigned
19	Q4N797	ATP-dependent RNA helicase (EC 3.6.4.13)	12	29.2	12.64 ± 1.32	Not assigned	ATP binding [GO:0005524]; hydrolase activity [GO:0016787]; RNA binding [GO:0003723]; RNA helicase activity [GO:0003724]
20	Q4N7W6	Rhoptry-associated protein	24	32.5	12.40 ± 1.34	Not assigned	Not assigned

PPI network analysis using the STRING database (v.12.0) identified corresponding *T. parva* orthologs for 17 of the top 20 proteins, with three proteins excluded due to the absence of identifiable orthologs (Supplementary Table S5). Of the 17 mapped orthologs, six formed an interaction network comprising five predicted functional associations, with an average node degree of 0.588 and an average local clustering coefficient of 0.314 (Fig. 3). Within the STRING network, the ATP-dependent RNA helicase (Q4N797) showed the highest number of predicted interactions (*n* = 3), with the pre-mRNA splicing factor protein (Q4N6I7), the zinc finger protein (Q4N7K8), and the *Sfi*I-subtelomeric related protein family member (Q4N8X8). All interactions were inferred from homologous protein evidence in other organisms.

**Fig. 3:**
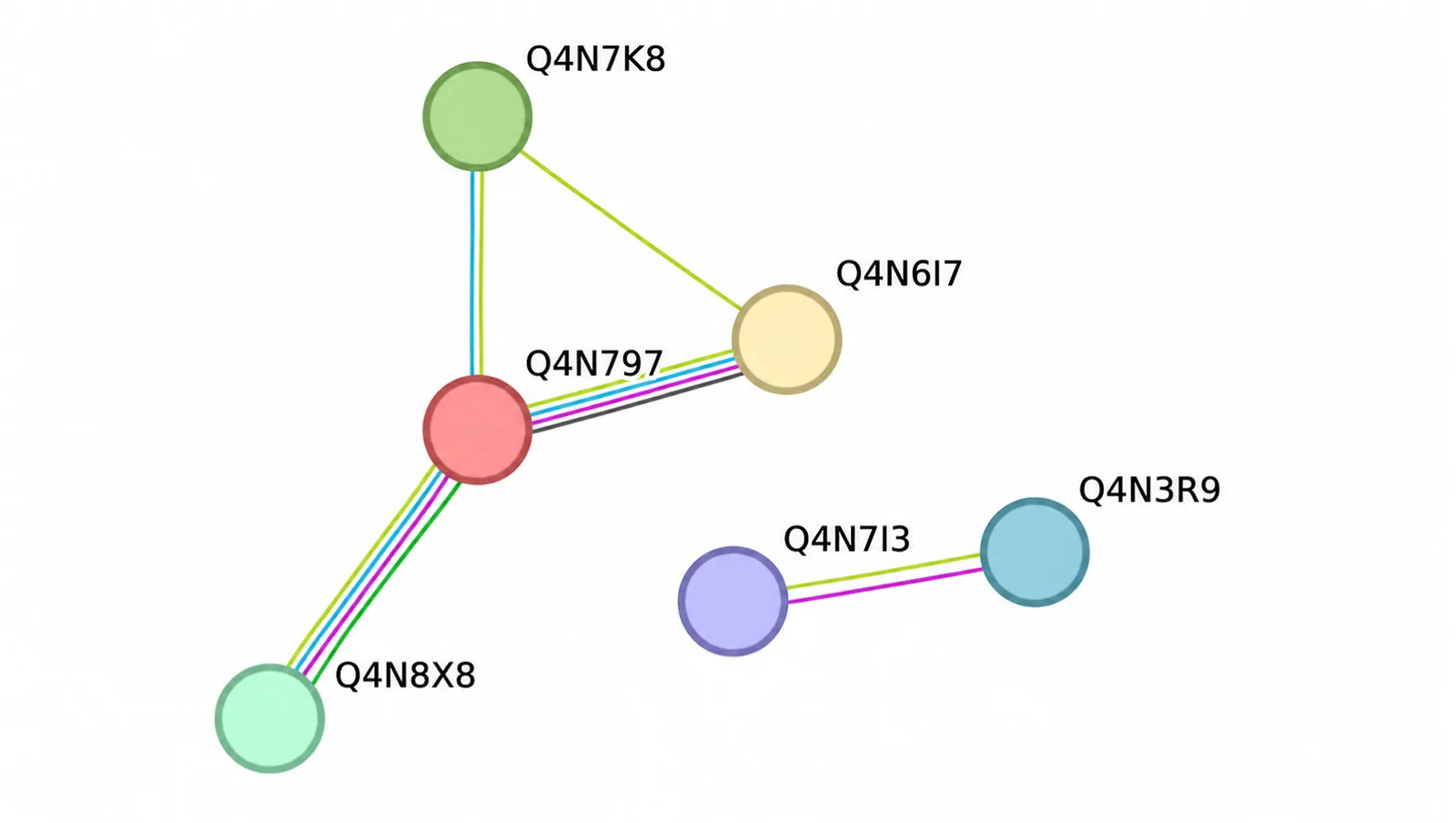
Protein-protein interaction network of the top 20 abundant *Theileria*-associated proteins identified in serum samples from the Fea’s muntjac (*Muntiacus feae*) and the northern red muntjac (*Muntiacus vaginalis*), constructed using the STRING database (v.12.0) with *Theileria parva* as the reference organism. Only proteins with predicted interactions are shown. Each node represents a protein, and edges indicate predicted functional associations based on evidence transferred from homologous proteins. The network comprised six nodes and five predicted functional associations. ATP-dependent RNA helicase (Q4N797) showed the highest number of predicted interactions (*n* = 3), with Q4N7K8, Q4N6I7, and Q4N8X8, whereas Q4N7I3 and Q4N3R9 formed a separate interaction pair. *Abbreviations*: Q4N3R9, E3 ubiquitin-protein ligase listerin; Q4N7I3, RING-type domain-containing protein; Q4N7K8, zinc finger protein; Q4N6I7, pre-mRNA splicing factor protein; Q4N797, ATP-dependent RNA helicase; Q4N8X8, *Sfi*I-subtelomeric related protein family member.

## 4 Discussion

This study provides integrative molecular and proteomic evidence of *Theileria* spp. infection in captive *M*. *feae* and *M*. *vaginalis* in Thailand. PCR screening identified *Theileria* spp. DNA in all examined animals, whereas *Babesia* spp. were below the detection limit of the PCR assay and *Anaplasmataceae* were not detected. The non-specific amplification observed in the *Babesia* spp. PCR may have reduced assay sensitivity for the intended target, particularly at low infection levels. Despite universal *Theileria* PCR positivity, no parasites were observed by blood smear examination, and all animals appeared clinically healthy, with hematological and blood chemistry parameters generally remaining within reference ranges. These findings are consistent with previous reports of *T. capreoli* infection in red deer (*Cervus elaphus*), *Theileria* spp. infection in white-tailed deer (*Odocoileus virginianus*), and *T. orientalis* infection in red deer in New Zealand, where infected animals were clinically normal, and PCR was more sensitive than microscopy for detecting low-level infections (Sawczuk et al., 2008; Noaman, 2012; Gieger et al., 2020; Lawrence et al., 2024). Additionally, our findings are consistent with subclinical infection and suggest a potential role of captive muntjacs in parasite maintenance, particularly in environments where competent vectors are present (Tiawsirisup et al., 2023; Sri-in et al., 2024).

Because all study animals except FM4F, a Fea’s muntjac of unrecorded origin, were born at their respective facilities, the detection of *Theileria* spp. DNA across all sampled facilities suggests that local vector exposure may contribute to transmission within these captive populations rather than infection having been acquired exclusively before captivity. This interpretation is consistent with previous observations at Khao Kheow Open Zoo, where *Rhipicephalus microplus*, *Dermacentor auratus*, and deer keds (*Lipoptena* spp.) have been directly documented and molecularly linked to *Theileria* spp., *Anaplasma* spp., and *Babesia* spp. (Tiawsirisup et al., 2023; Sri-in et al., 2024). At Chiang Mai Zoo and Om Koi Wildlife Breeding Center, no facility-specific vector surveys have been conducted, although *R. microplus* has been reported on red muntjac elsewhere in Chiang Mai Province (Sumrandee et al., 2016). Similarly, at Ton Nga Chang Wildlife Breeding Center, *Dermacentor* ticks have been recorded within the surrounding wildlife sanctuary, although they were found on non-cervid hosts and were not screened for pathogens (Apanaskevich et al., 2021). Therefore, the precise source and timing of infection cannot be determined, and dedicated vector surveillance at these three facilities remains an important area for future research.

Notably, universal *Theileria* positivity was observed despite routine in-feed ivermectin administration and weekly vegetation clearance at all four facilities, suggesting that these measures may not have completely prevented vector exposure or transmission. In contrast, *Babesia* spp. were below the detection limit of the PCR assay, and *Anaplasmataceae* were not detected. However, these findings do not exclude low-level infections, as pathogen loads may fall below the sensitivity of conventional PCR (Alvarez et al., 2019). In addition, the non-specific amplification observed in the *Babesia* spp. PCR may have further reduced assay sensitivity for the intended target. Phylogenetic analysis of the partial 18S rRNA sequences demonstrated genetic heterogeneity among the *Theileria* spp. sequences detected in the present study. Most isolates were positioned among *T. capreoli/T.* cf*. capreoli* reference sequences or other cervid-associated *Theileria* lineages, whereas MJ17M was positioned separately. Pairwise sequence identity analysis revealed high similarity within each set of isolates but greater divergence between them. Nevertheless, species-level interpretation should be made cautiously because relationships involving the study isolates were generally weakly supported. The relatively conserved nature of the 18S rRNA gene may provide insufficient variation for robust discrimination among closely related *Theileria* species or genotypes. Accordingly, proximity of a study sequence to a particular reference sequence in the phylogenetic tree should not, by itself, be considered definitive evidence of species identity when the corresponding relationship lacks bootstrap support. These findings nevertheless indicate genetic heterogeneity among the *Theileria* sequences detected in the study population. Further characterization using additional or more variable molecular markers, longer sequence regions, or multilocus approaches would help clarify their taxonomic identities and evolutionary relationships.

While PCR provides direct molecular evidence of pathogen DNA, serum proteomics provides complementary information on parasite-associated biological processes. Rather than serving as an additional diagnostic method, the proteomic analysis was performed to characterize parasite-associated proteins detected during naturally occurring subclinical infection. Among the most abundant proteins identified were rhoptry-associated proteins, aspartyl protease, calpain catalytic domain-containing proteins, E3 ubiquitin-protein ligases, RING-type domain-containing proteins, and ATP-dependent RNA helicase. Together, these proteins were associated with parasite invasion, proteolysis, ubiquitin-mediated regulation, and gene expression, consistent with parasite-associated biological processes during subclinical infection. Rhoptry-associated proteins play key roles in host cell invasion and effector delivery, whereas proteases such as aspartyl proteases and calpain-like proteins contribute to protein processing and parasite development (Roiko and Carruthers, 2009; Ben et al., 2021). Likewise, E3 ubiquitin-protein ligases and RING-type domain-containing proteins are involved in ubiquitin-mediated regulation, which has been implicated in protein turnover, signaling, and host cell modulation in apicomplexan parasites (Pickart and Eddins, 2004; Marsolier et al., 2015). ATP-dependent RNA helicases are generally associated with RNA processing and gene expression. These findings are consistent with biological processes associated with parasite activity and intracellular survival (Linder and Jankowsky, 2011; Woods et al., 2021; Buckingham and Ashby, 2022). Although the biological significance of these proteins in cervid-associated *Theileria* spp. requires further experimental validation, their detection provides preliminary functional insight into parasite-associated biological processes during naturally occurring subclinical infection.

Because the entire captive population of *M*. *feae* in Thailand currently consists of only seven individuals, the detection of *Theileria* spp. DNA in clinically healthy animals highlights the importance of incorporating routine molecular surveillance into conservation breeding and animal translocation programmes. Although all animals were clinically healthy, subclinical carriers may contribute to parasite maintenance where competent tick vectors are present. Regular PCR-based screening, together with tick surveillance and health monitoring, may therefore help minimize pathogen transmission within captive populations and reduce disease risks associated with conservation translocations, consistent with current international recommendations for wildlife disease surveillance and translocation planning (IUCN/SSC, 2013; WOAH, IUCN, 2024). A limitation of the present study is that conventional PCR followed by direct Sanger sequencing cannot reliably resolve mixed infections of closely related *Theileria* species. Future studies using lineage-resolving molecular approaches will be required to determine the frequency and composition of mixed infections in captive muntjacs.

## 5 Conclusion

This study demonstrates the molecular detection of *Theileria* spp. in clinically healthy captive *M*. *feae* and *M*. *vaginalis* in Thailand, consistent with subclinical *Theileria* spp. infections. Molecular and phylogenetic analyses showed that most isolates were positioned among *T. capreoli*/*T.* cf*. capreoli* reference sequences or other cervid-associated *Theileria* lineages, while one isolate was positioned separately; relationships involving the study isolates were generally weakly supported. Additionally, our findings support the potential role of captive muntjacs in parasite maintenance, particularly in environments where competent vectors are present. Serum proteomics complemented PCR-based detection by providing functional insight into parasite-associated proteins detected during subclinical infection. Although the biological roles of these proteins require further experimental validation, integrating molecular detection with proteomic profiling provides a useful framework for investigating parasite-associated biological processes and host-parasite interactions in wildlife.

## Ethical approval

All procedures were approved by the Chulalongkorn University Animal Care and Use Committee (CU-ACUC), Thailand (Approval No. 2131003).

## CRediT authorship contribution statement

**Pannawat Supapannachart:** Methodology, Investigation, Formal analysis, Data curation, Visualization, Funding acquisition, Writing - original draft, Writing - review & editing. **Sittiruk Roytrakul:** Conceptualization, Methodology, Validation, Formal analysis, Supervision, Writing - review & editing. **Narumon Phaonakrop:** Investigation, Formal analysis. **Thewarach Vechmanus:** Investigation, Resources. **Umaporn Maikaew:** Investigation, Resources. **Pridsana Sreepromma:** Investigation, Resources. **Karin Hirankailas:** Investigation, Resources. **Supatsorn Panyalert:** Investigation, Resources. **Thanapon Chotikaprakal:** Methodology, Investigation. **Sonthaya Tiawsirisup:** Methodology, Validation, Supervision. **Kritsada Thongmeesee:** Methodology, Investigation. **Rittidet Kongtawee:** Methodology, Investigation. **Piyanan Taweethavonsawat:** Conceptualization, Methodology, Validation, Supervision. **Gunnaporn Suriyaphol:** Conceptualization, Methodology, Validation, Formal analysis, Funding acquisition, Supervision, Writing - review & editing.

## Funding

This study was financially supported by the 10.13039/501100004704National Research Council of Thailand, the H.M. King Bhumibol Adulyadej 72nd Birthday Anniversary Scholarship from 10.13039/501100002873Chulalongkorn University, and the 90th Anniversary of 10.13039/501100002873Chulalongkorn University Scholarship (grant number GCUGR1125661059D). The funding bodies had no involvement in study design, data collection, data analysis, interpretation of results, or manuscript preparation.

## Declaration of competing interests

The authors declare that they have no known competing financial interests or personal relationships that could have appeared to influence the work reported in this paper.

## Data Availability

All data generated or analyzed during this study are included in this published article and its supplementary files. The newly generated sequences were submitted to the GenBank database under the accession numbers PZ295588-PZ295602. The LC-MS/MS proteomics datasets generated during this study are available in the ProteomeXchange repository under the accession IDs JPST004619 and PXD078238 (*via* jPOST: https://repository.jpostdb.org/preview/16214697446a0249346d810.access.key.7806).

## Appendix A Supplementary data

**Supplementary file 1**: Supplementary Table S1. Information for Fea’s muntjac (*Muntiacus feae*) and northern red muntjac (*Muntiacus vaginalis*) individuals included in the study. Supplementary Table S2. Hematological and blood chemistry profiles of Fea’s muntjac and northern red muntjac in this study. Supplementary Table S3. Pairwise nucleotide sequence identities (%) among *Theileria* 18S rRNA sequences obtained in this study and selected reference sequences from GenBank.Multimedia component 1

Supplementary file 2: Supplementary Table S4. Complete list of *Theileria*-associated proteins consistently detected in serum samples from Fea’s muntjac (*Muntiacus feae*) and northern red muntjac (*Muntiacus vaginalis*) using in-solution LC-MS/MS proteomics. Supplementary Table S5. Corresponding *Theileria parva* orthologs of the top 20 abundant *Theileria*-associated proteins identified for STRING-based protein-protein interaction (PPI) network analysis.Multimedia componet 2

Supplementary file 3: Supplementary Fig. S1. Representative agarose gel electrophoresis results for PCR detection of *Theileria* spp., *Anaplasmataceae*, and *Babesia* spp. in blood samples from Fea’s muntjac (*Muntiacus feae*) and northern red muntjac (*Muntiacus vaginalis*).Multimedia componet 3
