# Supplementary material for: Integrative molecular and serum proteomic insights into subclinical Theileria infection in captive Fea’s muntjac (Muntiacus feae) and red muntjac (Muntiacus vaginalis) in Thailand
Source: Curr Res Parasitol Vector Borne Dis. 2026 Sep 16;10:100437. doi: 10.1016/j.crpvbd.2026.100437 (PMC13633862; doi:10.1016/j.crpvbd.2026.100437)
Supplement: Supplementary file 1 [file mmc1.pdf]

Supplementary Table S1. Information for Fea's muntjac (*Muntiacus feae*) and northern red muntjac (*Muntiacus vaginalis*) individuals included in the study.

Sample ID	Animal ID	Sex	Age	Sampling location
FM1F	900113002103371	F	3 y 9 mo	Khao Kheow Open Zoo, ZPOT
FM2M	900113002103383	M	3 y 9 mo	Khao Kheow Open Zoo, ZPOT
FM3M	900113002103385	M	14 y 3 mo	Khao Kheow Open Zoo, ZPOT
FM4F	90006000007284	F	7 y 11 mo	Khao Kheow Open Zoo, ZPOT
FM6M	900012000840333	M	13 y	Chiangmai Zoo, ZPOT
FM7M	900012000568406	M	9 y 6 mo	Chiangmai Zoo, ZPOT
FM8M	TNDNP-01	M	16 y 3 mo	Ton Nga Chang Wildlife Breeding Center, DNP
MJ5M	31	M	N/D	Chiangmai Zoo, ZPOT
MJ8M	21	M	N/D	Chiangmai Zoo, ZPOT
MJ9M	22	M	N/D	Chiangmai Zoo, ZPOT
MJ10M	23	M	N/D	Chiangmai Zoo, ZPOT
MJ15F	MBD1	F	N/D	Om Koi Wildlife Breeding Center, DNP
MJ16F	MBD2	F	N/D	Om Koi Wildlife Breeding Center, DNP
MJ17M	MBD3	M	N/D	Om Koi Wildlife Breeding Center, DNP
MJ18F	MBD4	F	N/D	Om Koi Wildlife Breeding Center, DNP
MJ19F	MBD5	F	N/D	Om Koi Wildlife Breeding Center, DNP
MJ20F	MBD6	F	N/D	Om Koi Wildlife Breeding Center, DNP
MJ21M	MBD7	M	N/D	Om Koi Wildlife Breeding Center, DNP
MJ22M	MBD8	M	N/D	Om Koi Wildlife Breeding Center, DNP
MJ23M	MBD9	M	N/D	Om Koi Wildlife Breeding Center, DNP
MJ24F	MBD10	F	N/D	Om Koi Wildlife Breeding Center, DNP
MJ25M	MBD11	M	N/D	Om Koi Wildlife Breeding Center, DNP
MJ27F	MBD13	F	N/D	Om Koi Wildlife Breeding Center, DNP
MJ28M	BD002	M	N/D	Om Koi Wildlife Breeding Center, DNP
MJ29F	BD005	F	N/D	Om Koi Wildlife Breeding Center, DNP
MJ30M	BD006	M	N/D	Om Koi Wildlife Breeding Center, DNP
MJ31F	BD007	F	N/D	Om Koi Wildlife Breeding Center, DNP

Sample ID	Animal ID	Sex	Age	Sampling location
MJ32F	BD008	F	N/D	Om Koi Wildlife Breeding Center, DNP
MJ33F	OKB3	F	N/D	Om Koi Wildlife Breeding Center, DNP
MJ34M	OKB5	M	N/D	Om Koi Wildlife Breeding Center, DNP
MJ35F	OKB7	F	N/D	Om Koi Wildlife Breeding Center, DNP
MJ36M	MBD14	M	N/D	Om Koi Wildlife Breeding Center, DNP
MJ37F	MBD15	F	N/D	Om Koi Wildlife Breeding Center, DNP
MJ38F	BD001	F	N/D	Om Koi Wildlife Breeding Center, DNP

Abbreviations: DNP, Department of National Parks, Wildlife and Plant Conservation; F, female; M, male; N/D, not determined; ZPOT, Zoological Park Organization of Thailand under the Royal Patronage of H.M. the King.

Supplementary Table S2. Hematological and blood chemistry profiles of Fea's muntjac (*Muntiacus feae*) and northern red muntjac (*Muntiacus vaginalis*) in this study.

Parameter	Reference interval	Fea's muntjac (mean ± SEM)	Northern red muntjac (mean ± SEM)
Complete blood count			
RBC (×10^6 cells/mm^3)	3.55–19.81	17.94 ± 1.79	15.83 ± 0.53
Hemoglobin (g/dl)	8.7–20.4	14.35 ± 0.60	15.1 ± 0.46
Hematocrit (%)	24.3–57.3	43.00 ± 1.78	44.15 ± 1.40
MCV (fl)	22.3–80.6	24.60 ± 2.47	27.7 ± 0.53
MCHC (g/dl)	28.3–44.9	33.38 ± 0.05	34.26 ± 0.14
WBC (cells/mm^3)	1300–6600	3862.50 ± 283.12	2480 ± 210
Neutrophils (cells/mm^3)	483–3905	2391 ± 290.97	1676.44 ± 101.58
Band (cells/mm^3)	0–0.316	0 ± 0	0 ± 0
Lymphocytes (cells/mm^3)	355–3135	1296.00 ± 227.47	1314 ± 115.27
Monocytes (cells/mm^3)	19–474	59.00 ± 2.75	33.5 ± 2.75
Eosinophils (cells/mm^3)	0–218	67.50 ± 57.93	13.22 ± 7.07
Basophils (cells/mm^3)	0–132	42.25 ± 30.66	0 ± 0
Platelet (×10^3cells/mm^3)	66–1148	249 ± 36.17	412.27 ± 24.42
Blood chemistry			
BUN (mg/dl)	2.7–37.2	19.4 ± 1.8	26.54 ± 1.41
Creatinine (mg/dl)	1.09–3.07	2.26 ± 0.19	1.82 ± 0.06
AST (U/l)	12.0–77.0	51.7 ± 5.2	45.88 ± 11.89
ALP (U/l)	4.5–197.0	118.3 ± 11.6	111.08 ± 29.34
Total protein (g/dl)	5.67–8.0	7.00 ± 0.18	6.94 ± 0.19
Albumin (g/dl)	2.31–3.54	3.11 ± 0.24	3.84 ± 0.09

Abbreviations: ALP, alkaline phosphatase; AST, aspartate aminotransferase; BUN, blood urea nitrogen; MCHC, mean corpuscular hemoglobin concentration; MCV, mean corpuscular volume; RBC, red blood cells; SEM, standard error of the mean; WBC, white blood cells.

Supplementary Table S3. Pairwise nucleotide sequence identities (%) among *Theileria* 18S rRNA sequences obtained in this study and selected reference sequences from GenBank.

**Study isolate set/isolate**	**Study isolate codes**	**Within-group identity (%)**	**Closest GenBank reference(s)**	**Identity to reference(s) (%)**
T. capreoli-associated group	MJ23M, MJ29F, MJ27F, MJ37F, FM6M, MJ21M, MJ31F, MJ38F, MJ22M	99.79–100	*T. capreoli* (KJ451470, AY726011, PX916192); *T.* cf. *capreoli* (LC811654)	99.39–99.79
Other cervid *Theileria*-associated group	MJ18F, MJ28M, MJ33F, MJ16F, MJ24F	99.38–100	*T.* cf. *cervi* (LC811657); *Theileria* sp. Nara1 (LC623877); *Theileria* sp. Iwate 141 (AB602888)	98.35–98.76
MJ17M	MJ17M	–	*T.* cf. *capreoli* (LC811654)	98.76

**Comparison**	**Sequence identity (%)**
Isolates positioned among *T. capreoli/T.* cf. *capreoli* reference sequences *vs* isolates positioned among other cervid-associated *Theileria* lineages	97.53–97.94
MJ17M *vs T. capreoli*-associated group	98.35–98.56
MJ17M *vs* other cervid *Theileria*-associated group	98.55–98.96
