# Supplementary material for: Integrative molecular and serum proteomic insights into subclinical Theileria infection in captive Fea’s muntjac (Muntiacus feae) and red muntjac (Muntiacus vaginalis) in Thailand
Source: Curr Res Parasitol Vector Borne Dis. 2026 Sep 16;10:100437. doi: 10.1016/j.crpvbd.2026.100437 (PMC13633862; doi:10.1016/j.crpvbd.2026.100437)

Supplementary Figure S1. Representative agarose gel electrophoresis results for PCR detection of *Theileria* spp., *Anaplasmataceae*, and *Babesia* spp. in blood samples from the Fea's muntjac (*Muntiacus feae*) and the northern red muntjac (*Muntiacus vaginalis*). Sample IDs prefixed with FM and MJ indicate Fea's muntjac and northern red muntjac, respectively. M and F denote male and female, respectively. **A** PCR amplification of the 18S rRNA gene in *Theileria* spp. showing representative positive samples and the negative control. **B** PCR amplification of the 23S rRNA gene in *Anaplasmataceae* showing representative negative samples, the positive control, and the negative control. **C** PCR amplification of the 18S rRNA gene in *Babesia* spp. showing representative negative samples, the positive control, and the negative control. The additional bands observed in the sample lanes represent non-specific amplification products and were not considered positive *Babesia* spp. amplicons. *Key*: Lane M, DNA ladder; Lane P, positive control; Lane N, negative control. Expected amplicon sizes were approximately 1098 bp (*Theileria*), 830 bp (*Anaplasmataceae*), and 420–440 bp (*Babesia*).

A

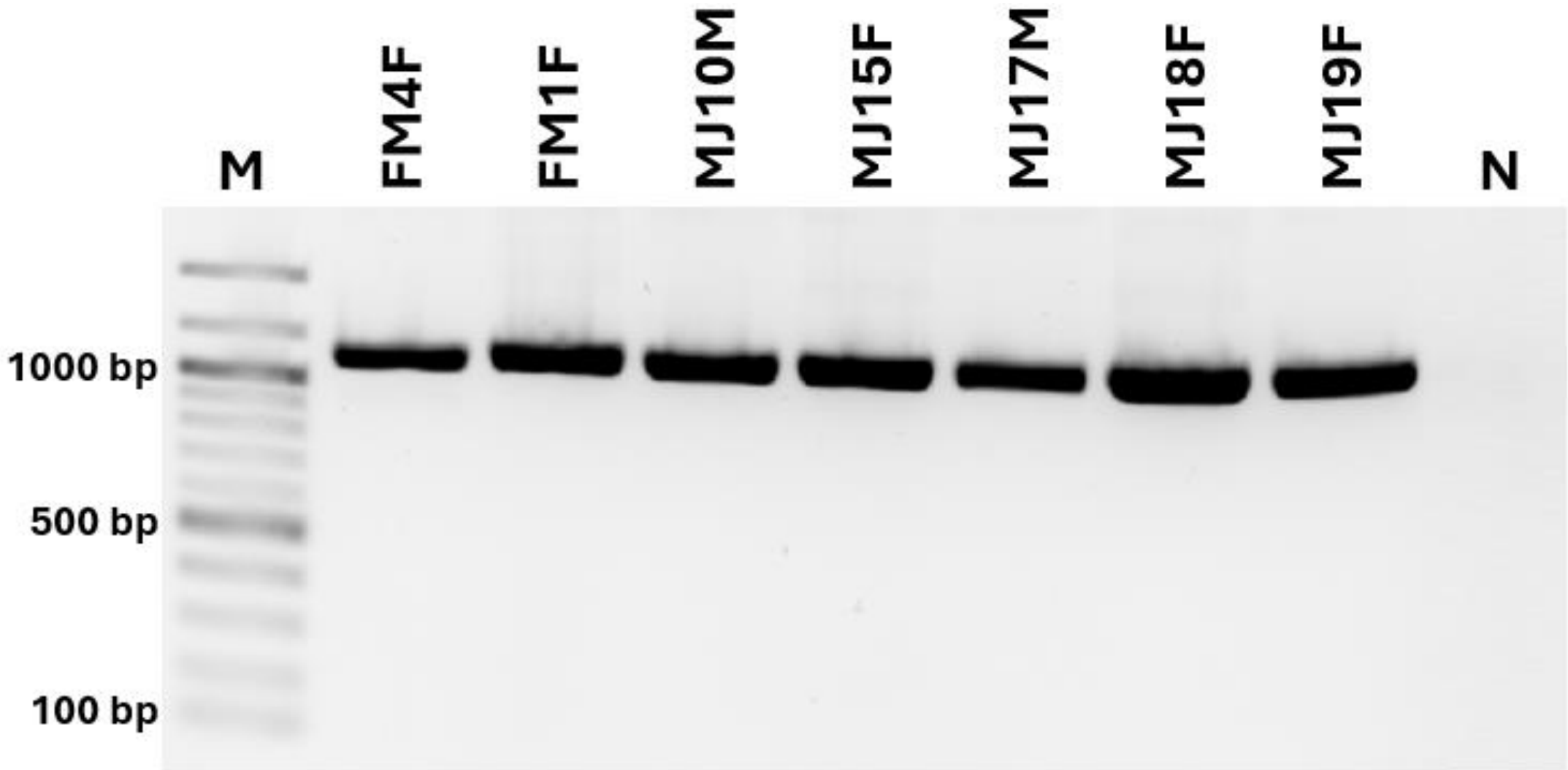

B

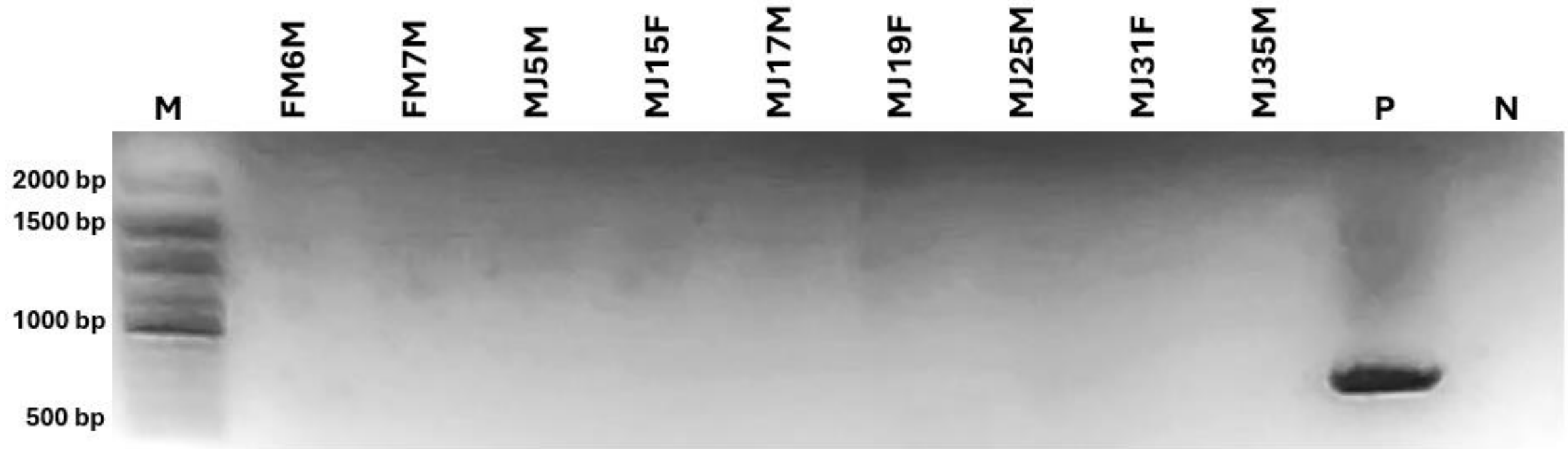

C

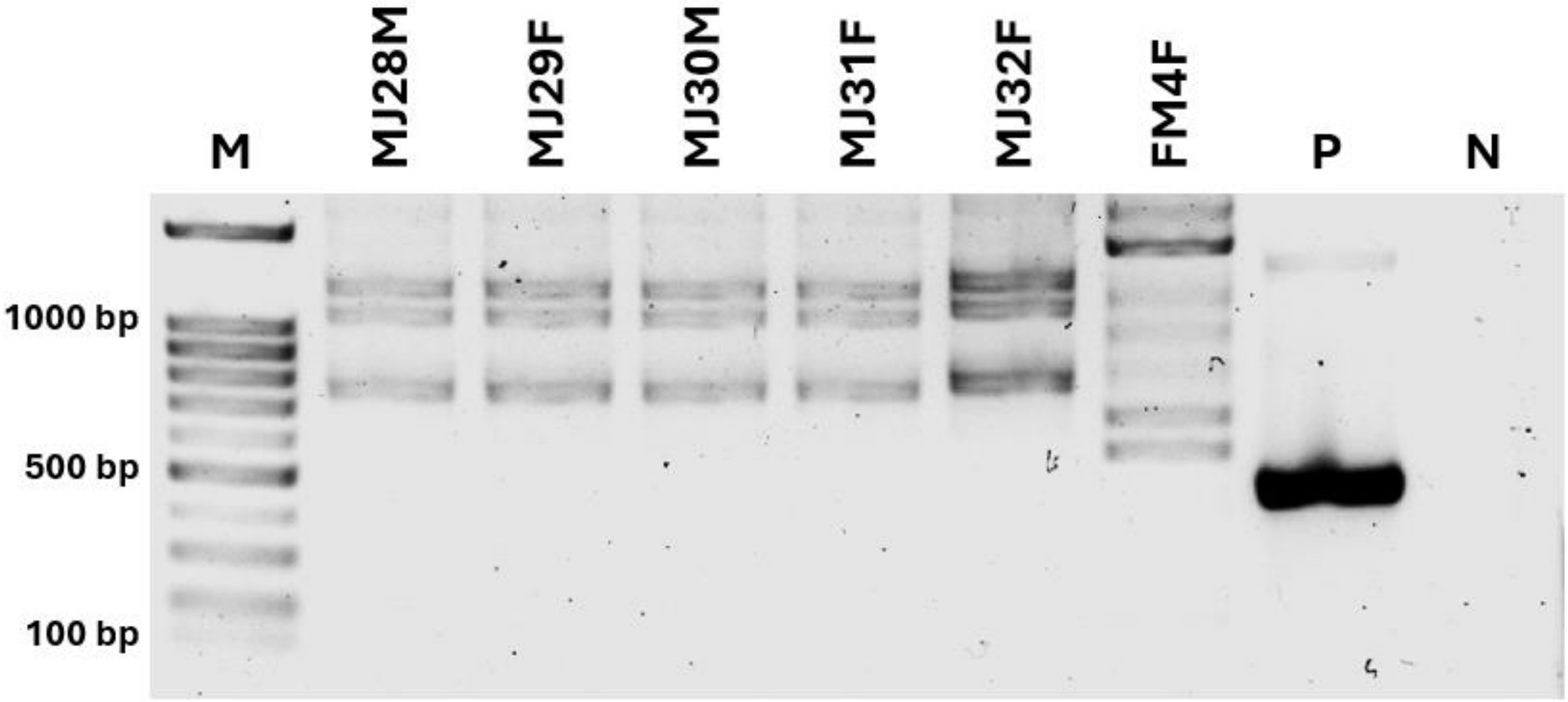

Supplement: Supplementary file 2 [file mmc3.pdf]
